# The importance of considering variability in re-expression of effect estimates for use in meta-analyses

**DOI:** 10.1186/s12874-025-02700-4

**Published:** 2025-10-30

**Authors:** Leonid Kopylev, Michael Dzierlenga

**Affiliations:** Center for Public Health and Environmental Assessment, Office of Research and Development, US Environmental Protection Agency, 1200 Pennsylvania Ave, Washington DC, NW 20460 USA

## Abstract

Comparing and combining reports from different publication is of interest to many when conducting meta-analyses. However, challenges can arise with reports using transformations of the exposure data. A recent publication, Linakis et al. (BMC Med Res Methodol 24:6, 2024), compared methods for re-expression with the conclusion that the re-expression methods examined are not reliable. In their analysis, they treated the estimated effect estimates, which are random variables, as if they were constants, which have no inherent variability. This letter describes two places where this assumption was made and how it affected their conclusions. While the re-expression methods demonstrate potential room for refinement in terms of estimating the observed point estimate, with the statistically appropriate consideration of variability, use of re-expression for small to moderate sample sizes (up to approximately 5000) seems appropriate. That contrasts with the author’s conclusion that use of re-expression methods is not suitable for meta-analyses.

## Letter

Linakis et al. [1] examined three methods for re-expression and found each to be unreliable. However, the authors oversimplified their analysis by treating the re-expressed estimate, $\:\widehat{{\beta\:}_{re-expressed}}$, and the reported estimate, $\:\widehat{{\beta\:}_{estimand}}$, as constants, when in fact they are random variables with inherent variability (note: the “hat” symbol represents the usual statistical notation for estimates). There are two instances where this assumption in made during the course of their analysis. This letter will outline both assumptions and discuss how the conclusions in Linakis et al. [1] were affected.

In the first instance, the lack of consideration of variability in the estimates affected the conclusions drawn from their Tables 4 and 5. In the investigation of the relative bias of $\:\widehat{{\beta\:}_{re-expressed}}$, the authors compared those values to $\:\widehat{{\beta\:}_{estimand}}$. Consideration of point estimates is important, but comparing point estimates of random variables ignores their variance, and ignoring variance may lead to unsupported conclusions. To address statistical variability, we added a two-sided confidence interval to $\:\widehat{{\beta\:}_{estimand}}$ (there is certainly more uncertainty around true beta: related to the fit of linear model, covariate structure, etc.) and to each of the three $\:\widehat{{\beta\:}_{re-expressed}\:}$ (Table 1), using the original code provided by Linakis et al. [1]. $\:\widehat{{\beta\:}_{estimand}}$ is quite variable, especially when the number of observations is low. We discuss the consequences of ignoring variability in the context of their Table 4, but the same applies to their Table 5 as well.

**Table 1:** Augmented table 4 from Linakis et al., manuscript. Each of column 2–5 has appropriate 95% confidence interval (CI) added. columns 6–8 show if an estimate $\:\widehat{{\beta\:}_{observed}}$ from column 2 is in corresponding confidence intervals in columns 3–5. columns 9–10 add variability of exposure and the size of the study. $\:\widehat{{\upsigma\:}}$ is an estimated standard deviation of the original exposure data, and n_obs_ is the study size. Y – yes, N- no

Author and year	$\:\widehat{{\beta\:}_{observed}}$with CI	$\:\widehat{{\beta\:}_{RB}}$with CI	$\:\widehat{{\beta\:}_{Dz}}$with CI	$\:\widehat{{\beta\:}_{Alt}}$with CI	Is$\:\widehat{{\beta\:}_{observed}}$in the CI for the corresponding$\:\widehat{{\beta\:}_{re-expressed}}$?			$\:\widehat{{\upsigma\:}}$	*n*_obs_
Abraham 2020 [2]	−0.064 (−0.11, −0.023)	−0.032 (−0.058, −0.0068)	−0.037 (−0.066, −0.0077)	−0.038 (−0.069, −0.008)	N	Y	Y	0.78	101
Lee 2020 [3]	1.44 (−0.37, 3.26)	1.31 (0.16, 2.47)	3.30 (0.53, 6.34)	3.46 (0.56, 6.66)	Y	Y	Y	0.83	124
Hamm 2010 [4]	1.5 (−7.6, 10.6)	3.7 (−5.1, 12.4)	3.9 (−5.4, 13.3)	3.9 (−5.3, 13.0)	Y	Y	Y	0.47	252
Apelberg 2007 [5]	−12.90 (−27.80, 2.00)	−11.70 (−25.10, 1.77)	−13.20 (−28.60, 1.92)	−13.20 (−28.60, 1.92)	Y	Y	Y	0.62	293
Washino 2009 [6]	−10.94 (−22.90, 1.10)	−11.40 (−22.70, −0.04)	−12.10 (−24.10, −0.04)	−10.00 (−20.10, −0.03)	Y	Y	Y	0.53	428
Chen 2012 [7]	−11.3 (−17.4, −5.2)	−16.3 (−26.0, −6.6)	−17.9 (−28.5, −7.2)	−17.8 (−28.4, −7.2)	Y	Y	Y	0.61	429
Pilkerton 2018 [8]	−0.0049 (−0.023, 0.014)	−0.021 (−0.041, −0.00033)	−0.03 (−0.06, −0.00065)	−0.031 (−0.061, −0.00066)	Y	Y	Y	0.55	621
Stein 2016 [9]	−0.0039 (−0.077, −0.0002)	−0.0068 (−0.013, −0.00013)	−0.0069 (−0.014, −0.000015)	−0.0069 (−0.014, −0.000015)	Y	Y	Y	0.52	1191
Darrow 2013 [10]	−2.30 (−4.80, 0.30)	−1.95 (−4.41, 0.51)	−2.02 (−4.61, 0.49)	−2.00 (−4.56, 0.48)	Y	Y	Y	0.54	1630
Xu 2020 [11]	0.51 (0.16, 0.81)	0.51 (0.26, 0.76)	0.96 (0.49, 1.44)	1.01 (0.51, 1.50)	Y	Y	Y	0.81	1947
Xu 2020 [11]	29.3 (14.1, 44.6)	25.4 (12.6, 38.2)	48.1 (23.9, 72.3)	50.4 (25.0, 75.7)	Y	Y	Y	0.81	1947
Cheang 2021 [12]	0.192 (0.042, 0.342)	0.335 (0.056, 0.614)	0.352 (0.059, 0.645)	0.353 (0.059, 0.647)	Y	Y	Y	0.47	2899
Odebeatu 2019 [13]	0.00069 (0.00065, 0.00073)	0.011 (0.0022, 0.021)	0.012 (0.0023, 0.022)	0.012 (0.0022, 0.021)	N	N	N	1.26	7765
Bulka 1987 [14]	0.015 (0.01, 0.021)	0.043 (0.021, 0.065)	0.051 (0.024, 0.077)	0.053 (0.025, 0.08)	N	N	N	0.75	8778
Steenland 2018 [15]	0.0011 (0.00093, 0.0012)	0.0012 (0.001, 0.0013)	0.0013 (0.0011, 0.0014)	0.0013 (0.0011, 0.0014)	Y	Y	Y	0.57	46,294

Table 1. Augmented Table 4 from Linakis et al., publication. Columns 2–5 have appropriate 95% two-sided confidence interval (CI) added. Columns 6–8 show if an estimate $\:\widehat{{\beta\:}_{observed}}$ from Column 2 is in corresponding confidence intervals in Columns 3–5. Columns 9–10 describe the variability of exposure and the size of the study.

There are several conclusions that could be made from evaluating if the confidence intervals of $\:\widehat{{\beta\:}_{re-expressed}}$ contain $\:\widehat{{\beta\:}_{estimand}}$. The first is that contrary to the original authors conclusions, size of $\:\widehat{{\upsigma\:}}$ (an estimated standard deviation of the exposure data) does not seem important. The size of the study seems to be a more important consideration. For all the small to moderate sample size studies examined, beta estimates obtained from the analysis of the original data are within the confidence intervals for corresponding re-expressed betas (except one case for one of re-expressed methods). For studies with very large sample sizes (greater than approximately 5,000), $\:\widehat{{\beta\:}_{estimand}}$ is more precise and is more likely to fall outside the confidence interval of the re-expressed effect estimate. In this case, only one out of three studies with very large sample sizes is consistent with the re-expressed values. For the other two large studies, the confidence interval around $\:\widehat{{\beta\:}_{estimand}}$ does not intersect with confidence intervals around $\:\widehat{{\beta\:}_{re-expressed}}$ indicating statistically significant differences. Considering statistical significance, re-expression of studies with small to moderate sample sizes seems appropriate, rather than the conclusion of Linakis et al. [1] that re-expression methods are not suitable. Studies with sample sizes above 5000 are relatively rare, so re-expression would be applicable to most of the epidemiologic literature.

In the second instance, variability was also not appropriately considered in the Linakis et al. [1] Monte Carlo simulations designed to investigate coverage of the re-expressed estimates. The simulations considered $\:\widehat{{\beta\:}_{estimand}}$, as the Monte Carlo estimand. The authors did this to investigate “… performance of the estimator(s) in specific datasets.” While their reasoning is understandable, there is no theoretical basis (e.g., Morris et al. [16] for using a random variable (and not a known constant – a true value) as a target of Monte Carlo simulations. We present a simulation to illustrate the variability in $\:\widehat{{\beta\:}_{estimand}}$ and thereby demonstrate the fact that the Linakis et al. [1] Monte Carlo simulation targeted a random variable, not a known constant. Our simulation involved 2000 iterations for each pair of n_obs_ and $\:\sigma\:$, where n_obs_ is the sample size the authors considered (162 or 8484) and $\:\sigma\:$ is the standard deviation of the log-transformed data (0.25, 0.45, 0.65, 0.85).

Table 2 confirms that for n_obs_=162 the variability is too large for $\:\widehat{{\beta\:}_{estimand}}$ to be an appropriate target for Monte Carlo (normally, the target would be a constant), because the variance of $\:\widehat{{\beta\:}_{estimand}}$ does not decrease with the number of Monte Carlo simulations. Interestingly, the variability decreases with increasing σ. This presents the question of whether the Linakis et al. [1] finding of interaction between the re-expression method performance, defined essentially as the ratio $\:\widehat{{\beta\:}_{re-expressed}}$ / $\:\widehat{{\beta\:}_{estimand}}$, and σ is affected by the trend in variability. Even for n_obs_= 8474 the variability can be as much as 15% each direction for smaller σ.

**Table 2:** Selected results of simulations showing variability in $\:\widehat{{\beta\:}_{estimand}}$. In this scenario: number of simulations = 2000, Logbase = 10, $\:{\beta\:}_{DGM}=1$, and Median=8. Lower % shows 5th percentile of Monte Carlo sample of $\:\widehat{{\beta\:}_{estimand}}$, as percent of mean( $\:\widehat{{\beta\:}_{estimand}}$). Upper % shows the same for 95th percentile

*n*_obs_	$\:{\upsigma\:}$	Lower %	Upper %
162	0.25	−110.7	113.7
	0.45	−67.4	67.8
	0.65	−50.2	55.8
	0.85	−48.7	53.3
8474	0.25	−15.5	15.3
	0.45	−9.3	9.1
	0.65	−7.8	7.4
	0.85	−9.3	9.0

To properly evaluate the re-expression methods, the authors should use a constant parameter as the target for the re-expressed effect estimates. That will assure theoretical validity and avoid the variability issues illustrated here. Linakis et al. [1] generated data with known $\:{\beta\:}_{DGM}\:$(DGM for data generating mechanism) in logged space (i.e., the outcome was specified to be proportional to the logarithm of the generated exposure distribution). Instead of that, generating data with $\:{\beta\:}_{DGM}$ in un-logged space (i.e. specifying the outcome as proportional to the generated exposure distribution without log transformation) resolves the issue.

The analysis presented in this letter indicates the importance of considering statistical variability in comparing between random variables and in the design of Monte Carlo simulations. When statistical variability was considered in the evaluation of re-expression methods using real data, the re-expression methods produced values with no difference, in terms of statistical significance, from the observed effect estimate in most cases. When there was a significant difference, this was generally attributable to an observed effect estimate that was precise as a result of a large sample size (>5,000). There was no apparent effect due to the width of the exposure distribution, as was suggested by the original analysis of the simulated data. For the Monte Carlo analysis, comparing the re-expressed effect estimates to an effect estimate which was variable produced results that were difficult to interpret because the amount of variability in the effect estimate depends on the parameters used. Because of this, the conclusions regarding how parameters affect the performance of the re-expression are unclear. This issue can be avoided by designing a Monte Carlo analysis for which the target of the simulation is a known constant, which requires a relatively simple redesign of the study, in this case.

The additional analysis we have performed is limited. While we conducted a supplemental analysis to include variability in the empirical data that was previously examined by Linakis et al. [1], additional validation would be needed to confirm our findings. Furthermore, while we believe there are theoretical issues with the use of a random variable as a target in Monte Carlo and that this is explanatory for the high dependence of re-expression performance on σ, we did not replicate the original study with the modified Monte Carlo design to confirm this hypothesis. Despite these limitations, we thought that these considerations were substantial enough to publish so that others considering the potential application of these methods would be informed of them.

The decision to use any re-expression method is necessarily a compromise between a more comprehensive meta-analysis with additional uncertainty introduced by re-expression, and a less comprehensive meta-analysis that focuses only on studies with similar analytical approaches. While re-expression methods demonstrate potential room for refinement in terms of estimating the observed point estimate, we found that, in most cases, re-expressed values were not significantly different from the observed value.

## Data Availability

Data is provided within the manuscript.
